# The relation between preoperative radiological sarcopenia and postoperative recovery of physical activity in older surgical cancer patients; an explorative study

**DOI:** 10.1016/j.jnha.2024.100345

**Published:** 2024-08-24

**Authors:** S. Hendriks, M.G. Huisman, L. Weerink, L.T. Jonker, B.C. van Munster, J.J. de Haan, G.H. de Bock, B.L. van Leeuwen

**Affiliations:** a Department of Surgery, University of Groningen, University Medical Center Groningen, Groningen, The Netherlands; b Department of Internal Medicine, University Medical Center Groningen, Groningen, The Netherlands; c Department of Medical Oncology, University of Groningen, University Medical Center Groningen, Groningen, The Netherlands; d Department of Epidemiology, University of Groningen, University Medical Center Groningen, Groningen, The Netherlands

**Keywords:** Older adults, Surgical oncology, Radiological sarcopenia, Physical activity, Independent living

## Abstract

To identify older surgical cancer patients at risk of decreased postoperative recovery of physical activity (PA), this study assesses whether preoperative radiological sarcopenia (RS) is associated with a decreased ability to return to baseline PA. RS was defined as decreased psoas muscle mass or -density by gender-specific cut-offs on CT-scans at level of vertebra L3. PA was assessed as steps/day measured with PA tracker and recovery of PA was defined as >90% of preoperative steps/day at 3 months postoperatively. Of 44 included patients aged 65 and over undergoing oncologic surgery, 18 patients (41%) showed RS. Seventeen patients (39%) returned to baseline PA, of which eight patients had RS (47%). RS was not associated with a return to baseline PA (OR: 1.38, 95%CI 0.39–4.92, p = 0.61). In this exploratory study, no association was found between preoperative RS and recovery of PA postoperatively.

## Highlights

• 41% of older oncological surgical patients showed RS preoperatively.
• 39% of the patients recovered to ≥90% of preoperative physical activity.
• 47% of the patients with RS recovered to ≥90% of preoperative physical activity.
• RS is not associated with postoperative recovery of physical activity.

## Graphical abstract

**Figure:**
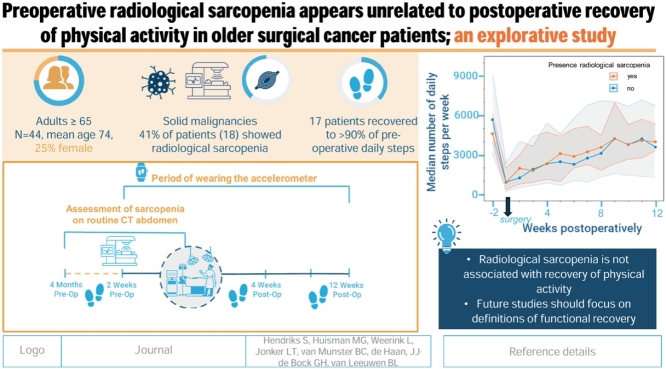

## 1 Introduction

With the ageing population, the global burden of older adults with cancer is rapidly increasing. The number of new cancer cases in older adults is expected to double by 2035 compared to 2012 [1]. For solid types of cancer, which predominantly affect older adults, surgery remains the cornerstone of treatment [2]. Maintaining independence is often an important treatment goal for older patients, even more so than survival itself [3].

In Europe, the prevalence of sarcopenia in community-dwelling older people ranges from 10% to 27% and might be even higher in patients with cancer [4]. Sarcopenia is defined as a progressive and generalized skeletal muscle disease that is associated with an increased likelihood of adverse outcomes, such as falls, physical disability and mortality [5]. In older adults undergoing oncologic surgery, the presence of radiological sarcopenia preoperatively has been associated with negative outcomes such as an increased risk of major postoperative complications, longer hospital stay and extended intensive care admittance [[6], [7], [8]]. In addition, recently pre-operatively measured sarcopenia was shown to be an independent predictor of 90-day mortality in older adults undergoing non-cardiac thoracic – or abdominal surgery [9]. Adverse outcomes, such as complications, negatively affect postoperative recovery and physical activity and – functioning [[10], [11], [12]].

Certain levels of physical activity and – functioning are needed to perform ADL-tasks independently [13]. As physical activity and - functioning is decreased, this might result in a decline in independence in daily living, which is an important outcome for older patients. Sarcopenia itself is associated with reduced physical activity and poor quality of life in community dwelling older adults [14,15]. In colorectal surgery, preoperative diagnosis of sarcopenia is related to functional outcomes postoperatively [16]. We hypothesize that when sarcopenia is diagnosed preoperatively, this might be associated with a greater decline in physical activity postoperatively. This could on its turn lead to loss of independence [13].

Psoas muscle mass evaluated on CT-scans was shown to be an important predictor of development of severe postoperative complications and 30-day mortality in cancer patients going for surgery for solid malignancies [17]. Objective physical activity can easily be measured by wearable accelerometers, which are able to capture objective data over longer periods of time, only by being worn by the patient [18]. In cancer patients, measuring physical activity using accelerometers is easy to use in clinical patients and accelerometers were able to identify cancer patients at risk for readmission [19,20]. Also, after major surgery, accelerometers have been shown to be easy and practical in monitoring physical activity, even in older patients [10,21].

Higher levels of physical activity, such as steps/day, are associated with better performance in Activities of Daily Living (ADL). Good ADL performance is needed in maintaining independence, often an important treatment goal [[22], [23], [24]]. Moderate-to-vigorous physical activity (MVPA) is also measured by acetometers is associated with skeletal muscle strength and - power and better ADL performance [13]. As recovery of physical activity and regaining independence are important outcomes for older cancer patients, being able to inform patients preoperatively about these outcomes can be very helpful in the decision-making process preoperatively [22,23]. Also, knowing which patients are at risk of a longer recovery period and/or prolonged dependence might be beneficial in providing the right care in time. Therefore, the main objective of this exploratory study was to investigate whether preoperative RS was associated with a return to preoperative physical activity until 3 months postoperatively, in steps/day. In addition, the recovery of physical activity as steps/day and MVPA and its association with RS were explored.

## 2 Material and methods

### 2.1 Study design

This exploratory study analyzed data from a single-center observational cohort study, that was performed as part of the *Connecare* research consortium [10]. As studies on recovery of physical activity and regaining independence in older adults with cancer undergoing surgery are lacking and data collection is laboursome, an exploratory design was chosen [25]. The cohort study was conducted in the University Medical Center Groningen (UMCG). The study was registered in the Dutch clinical Trial Database (registration number: NL8253) and was approved by the medical ethics committee of the UMCG. Data collection was conducted according to the revised version of the Declaration of Helsinki (October 2013, Brazil). Patients were enrolled between May 2018 and July 2019. Patients in this cohort study were described earlier [10,26,27].

### 2.2 Patients and clinical data collection

For the cohort study, patients aged 65 year and over who were scheduled for surgery for a solid malignant tumor were included [10]. Exclusion criteria were non-elective surgery, the use of walking aids, being wheelchair- or bed-ridden, and severe limitation in hearing, vision, and/or cognition that were expected to impair the patient’s ability to read the tablet, consult by telephone, or understand how to synchronize wearables with the tablet [10]. In the current study, patients were included in the analysis if their step count data measured with wearable physical activity tracker was available preoperatively and at least consecutively the first month postoperatively. Availability of a routine CT-scan within 4 months preoperatively for sarcopenia measurements was required (Supplementary Figure S1).

### 2.3 Endpoints

The primary endpoint of this exploratory study was the return to baseline physical activity in steps/day three months postoperatively defined as ≥90% of preoperatively steps per day [10]. Secondary endpoints were the number of steps/day one month postoperatively, the MVPA (minutes/day) at one and three months postoperatively, the Timed Up and Go (TUG) test at three months postoperatively and trends in the recovery of physical activity when plotting the consecutive physical activity 3 months postoperatively (Supplementary Figure S1). Besides physical activity, other endpoints were 30-day complications and length of hospital stay.

### 2.4 Radiological sarcopenia measurement

The presence of RS was assessed using CT imaging. Computed Tomography (CT) is considered the gold standard to radiologically assess muscle mass and quality [28]. Identifying sarcopenia on a CT scan routinely obtained in the oncological workup, i.e., radiological sarcopenia (RS), does not require additional tests or interventions for the patient [17]. All CT images were obtained 60–70 seconds after the administration of intravenous iodinated contrast media. The muscle mass was assessed with use of the Total Psoas Area (TPA, cm^2^) determined by delineating both psoas muscles and calculation of the surface area. The TPA was adjusted for the patient’s length to calculate the Total Psoas Index (TPI, cm^2^/m^2^) [29]. Subsequently, the muscle quality was evaluated with assessment of the radiodensity (Hounsfield Units, Hus) of the psoas muscles (TPRD). This was automatically done on the area selected for surface measurement. The foremost cranial slice clearly showing both transverse process of the L3 vertebra was used for the measurement. The thinnest available slice thickness, generally 1 mm, was used. The delineating of the psoas muscles was performed manually by a radiologist (LW) with use of imaging analysis software (Aquarius Intuition, Terarecon Inc., Foster City, CA) [[30], [31], [32]].

Based on previous studies together with a large comparable cohort study including patients undergoing surgery for solid malignancies, sarcopenia was defined as low psoas muscle area or low psoas muscle quality on CT abdomen. The gender specific cutoff values for low psoas muscle mass were TPI < 5.9 cm^2^/m^2^ in males and TPI < 4.7 cm^2^/m^2^ in females [32]. Based on this cohort, for low muscle quality the gender specific cutoff values were TPRD < 41.5 HU in males and <42.8 HU in females

### 2.5 Physical activity measurement

In this study the accelerometer-based wearable activity monitor used was the Fitbit Charge 2 (Fitbit Inc., San Francisco, CA), which uses an accelerometer to capture body motion in three-dimensional space [33]. This accelerometer is able to capture large amounts of data over longer periods of time, easy to administer and objectively measures physical activity [18]. The Fitbit data values that were used for analysis were daily step count and time spent in MVPA [10]. If data was missing or if physical activity was very low (<1000 steps/day if 1000-steps/day had been achieved in the previous days was considered missing data for that specific day), patients were contacted to provide assistance in synchronization of the wearable or to detect clinically relevant reasons for low physical activity levels [10]. These were measured in 24-h periods from two weeks before surgery until three months postoperatively consecutively. MVPA was defined as the minutes per day spent on activities with an intensity of ≥3 Metabolic Equivalent of Tasks [34]. The Fitbit was provided at the preoperative assessment and worn on the patient’s non-dominant wrist all days of study participation except during surgery, IC admission, bathing/showering and battery charging. Step count was visible to the patient, though no step goal was provided.

Steps/day and MVPA/day per time were consecutively measured until three months postoperatively and analyzed as median values per week. For example, steps/day or MVPA/day at one month was defined as median value in the 4th week postoperative. If the 4th week was not available, the 5th week was taken. Steps/day or MVPA/day at three months was defined as median values in the 12th week postoperative. If the 12th week was not available, the week before or after the 12th week was taken.

### 2.6 Data collection and definitions

Clinical data, such as age, sex, BMI, tumor type, disease stage, comorbidities, and surgical characteristics were prospectively collected from the patient’s medical record. Patients’ baseline characteristics were collected preoperatively, at home or at admission, including preoperative physical status assessment using the American Society of Anesthesiologist (ASA) scores, ADL- and instrumental ADL (iADL) scores, hand grip strength tests and the TUG test. Complications up to 30 days postoperatively were recorded prospectively and collected, classifying complications by Clavien–Dindo scores [35].

### 2.7 Statistical analysis

As the nature of this study was explorative, no sample size calculation was performed. We used all available data. Descriptive data were reported as absolute numbers and as percentages for categorical data. For continuous data, distributions were analyzed and mean and standard deviation (SD) or median and interquartile range (IQR) were given where appropriate. Baseline -, surgical, and physical activity characteristics were compared between two groups, patients with RS and patients without RS. Continues parametric data, non-parametric data and categorial data form these two groups were compared using the independent T-test, Mann–Whitney U test and Fisher’s exact test where appropriate.

Mann-Whitney U test was used to compare steps/day and the MVPA at one- and three months postoperatively and the TUG at three months postoperatively for patients with RS and patients without RS. For the association of the presence of sarcopenia and return to baseline physical activity, a logistic regression analysis was performed.

To get a better insight in the patterns of recovery of steps/day, median steps/day per week (together with the range (IQR)) were plotted for patients with sarcopenia against patients without sarcopenia. These graphics were also made for median MVPA in minutes/day per month. A sensitivity analysis was performed after exclusion of patients with extra-cavitary types of cancer, such as skin- or vulva cancer, as RS might manifest differently in these groups. Analysis for steps/day and MVPA/day were analyzed in this group as above mentioned.

## 3 Results

### 3.1 Patient and disease characteristics

In total, 44 patients were included in this study with a mean age of 73.6 years (SD 4.9), and 11 patients were female (25%). Most patients underwent surgery for colorectal cancer (n = 31, 71%). In three patients, the tumor was located in the stomach (7%), other sites were vulva (7%), skin (5%), liver or biliary tract (2%), soft tissue (2%) or kidney (2%) (Table 1). Eighteen patients were sarcopenic (41%). Clinical data and surgical characteristics did not differ when comparing patients without RS to patients with RS, apart from BMI. Patients without RS had a BMI of 28.5 (SD 3.9) which differed from 26.0 (SD 3.4) for patients with RS (P < 0.05).

**Table 1:** Characteristics of patients (n = 44).

Patient characteristics	
Age, mean, SD	73.6 (4.9)
Sex	
Female	11 (25.0%)
Male	33 (75.0%)
ASA classification	
ASA 1	2 (4.5%)
ASA 2	33 (75.0%)
ASA 3	9 (20.5%)
CCI score, median [IQR]	3.5 [2.0−6.0]
BMI, mean (SD)	27.5 (3.8)
<25 kg/m^2^	13 (29.5%)
≥25 kg/m^2^	31 (70.5%)
Preoperative functional assessment scores	
ADL, median [IQR]	0 [0−0]
>0	7 (15.9%)
iADL, median [IQR]	8.0 [7.5−8.0]
<8	11 (25.0%)
TUG (seconds)	8.2 [7.4−9.7
Hand grip strength (dominant hand, kg)	
Female	24.3 (4.8)
Male	38.0 (9.3)
Tumor- and surgical variables	
Tumor location	
Colon/rectum	31 (70.5%)
Gastric	3 (6.8%)
Vulva	3 (6.8%)
Skin	3 (6.8%)
Hepato-biliary	1 (2.3%)
Soft tissue	1 (2.3%)
Renal	1 (2.3%)
Type of surgery	
Open/converted	33 (75.0%)
Laparoscopy/robot/hybrid	11 (25.0%)
Procedure time (minutes), median [IQR]	393 [211−523]
Length hospital stay (days), median [IQR]	8.5 [5.0−14.5]
Any complications during admission	
No	26 (59.1%)
Yes	18 (40.9%)

SD, Standard Deviation; ASA, American Society of Anesthesiologist; IQR, Interquartile range; CCI, Charlson Comorbidities Index; BMI, Body Mass Index; (i)ADL, (instrumental) Activities of Daily Living; TUG, Timed Up and Go test.

### 3.2 Radiological sarcopenia and physical activity

Step count data at one month postoperatively was available for all patients, in 40 patients (91%) step count was available three months postoperatively. Baseline steps did not differ between groups, with a median of 4374 steps/day (IQR 3225−6568) for patients with RS and 5762 steps/day (IQR 4772−7929) for patients without RS (p = 0.18) (Table 2). Baseline MVPA also did not differ between RS- and non-RS patients, median MVPA for patients with RS was 30 min/day [IQR 10−58]. For patients without RS, the median MVPA was 38 min/day [IQR 18−65] (p = 0.34). Preoperative TUG test results did not differ between RS- and non-RS patients with a median of 8.2 s for patients with RS [IQR 6.7–9.8] and 8.3 s for patients without RS [IQR 7.6–9.7] (p = 0,58). In the sensitivity analysis, 39 patients with intracavitary types of cancer were analyzed. Patients with intra-cavitary types of cancer did not show significant difference with regard to baseline- and postoperative steps/day and MVPA (at one and three months postoperatively, Supplementary Table S1).

**Table 2:** Physical activity pre- and postoperatively stratified by the absence or presence of radiological sarcopenia.

Steps per day: median [IQR]			
	Non-radiologic sarcopenic (n = 26)	Radiologic Sarcopenic(n = 18)	p-value^+^
Baseline	5762 [4772−7929]	4374 [3225−6568]	0.18
At 1 month* postoperatively	2337 [870−5418]	2330 [1160−4749]	0.95
At 3 months*	3603 [1287−6810]	3999 [3362−5329]	0.69
% of baseline at 3 months*	79.9 [50.4−100.9]	88.0 [77.2−125.2]	0.08
Recovered# at 3 months			
Yes	9 (39.1%)	8 (47.1%)	0.61
No	14 (60.9%)	9 (52.9%)	
MPVA in minutes/day, median [IQR])			
Baseline	38 [18−65]	30 [10−58]	0.34
MVPA 1 month*	15 [3–45]	14 [8–33]	0.96
MVPA 3 months*	26 [11−67]	25 [12–53]	0.99
% of baseline at 3 months*	69.8 [44.3−126.2]	88.1 [51.6−147.1]	0.33
Recovered# at 3 months			
Yes	7 (31.8%)	5 (41.7%)	0.57
No	15 (68.2%)	7 (58.3%)	

IQR, Interquartile range; MVPA, Moderate – Vigorous Physical Activity.

*Postoperatively, ^#^recovered = >90% of baseline value.

^+^Values for patients with- and without radiologic sarcopenia were testes using Mann Whitney U Tests.

At three months postoperatively, in 17 patients (39%) the number of daily steps returned to >90% of baseline level. Of these 17 patients, eight patients had RS (47%) and nine patients did not (39%). RS was not associated with the ability to return to baseline number of daily steps (OR 1.38, 95%CI 0.39–4.92, p = 0.61).

The absolute number of steps one month postoperatively did not differ between groups with 2330 steps/day (IQR 1160−4749) for patients with RS and 2337 steps/day (IQR 870−5418) for patients without RS (p = 0.95). At three months, the number of steps/day was 3999 steps/day (IQR 3362−5329) and 3603 steps/day (IQR 1287−6810), for patients with and without RS respectively (p = 0.69, Table 2). MVPA in minutes/day did also not differ at one- and three months postoperatively for RS and non-RS patients (Table 2). The difference in MVPA per week at three months as percentage of baseline was also not statistically significant (88% vs 70% respectively, p = 0.33).

TUG test results three months postoperatively was performed by 35 patients and did not differ between groups, with a median of 7.9 s for patients with RS [IQR 6.8–10.3] and 8.4 s for patients without RS [IQR 7.4–9.5] (p = 0,44). Length of hospital stay did not differ between groups.

### 3.3 Radiological sarcopenia and postoperative outcomes

In total, 18 patients experienced complications (41%, Table 1), of which 13 were in the group of patients without RS. Readmissions occurred in three (17%) patients with the presence of RS and in seven (27%) patients without RS (p = 0.40).

## 4 Discussion

This exploratory study is the first providing insight in preoperative RS and postoperative objectively measured recovery related to physical activity in a small but well-defined cohort. Of the 44 included patients aged 65 and over undergoing oncologic surgery, 17 patients (39%) returned three months postoperatively to baseline steps/day, of which eight patients had RS (47%), and nine patients did not (39%). RS was not associated with the ability to return to baseline number of daily steps.

In the general older population, higher levels of physical activity protect against the occurrence of sarcopenia [36]. Specifically, associations have been shown between the number of daily steps, MVPA and occurrence of sarcopenia [[23], [24], [25]]. Meier et al., observed that sarcopenic older adults walked 4063 steps/day vs. 5050 steps/day for non-sarcopenic older adults (p 0.04) [37]. In our cohort, no significant difference in median baseline steps/day was found in sarcopenic vs. non-sarcopenic patients or time spent in MVPA, which is mainly explained by our small sample size. The number of steps per day at baseline for both sarcopenic and non-sarcopenic older surgical cancer patients were similar to two large European cohorts in community-dwelling older adults [38,39]. This might indicate that for patients undergoing treatment for malignancies, they might be relatively active. Westbury et al. found that lower levels of physical activity, including time spent in MVPA and in non-sedentary physical activity levels in minutes/day were associated with sarcopenia. Median daily time spent in MVPA was only 14.3 min for men and 9.5 min for women [40]. Our patients spent more time in MVPA, with median daily MVPA levels of 30 min/day for patients with RS and 38 min/day [IQR 18−65] for patients without RS. The higher mean age of patients in the study of Westbury et al. of 78 years, versus the median age of 73 years of the present study could party explain the reported difference in physical activity. This difference can also be attributed to the fact that we have included a small sample size and investigated older patients with cancer, whereas the preceding studies included community-dwelling older adults.

Our hypothesis was that preoperative RS is related to diminished abilities to physical recovery after surgery. This relation was not shown in the present study, which could indicate that this relation is indeed not strong, but could also be a result of our small sample size. Of the 44 included patients aged 65 and over, undergoing oncologic surgery, 17 patients (39%) returned three months postoperatively to baseline physical activity, of which eight patients had RS (47%), and nine patients did not (39%). This is an important finding, as it implicates that it will take a long time after oncologic surgery to return to baseline physical activity, also for those without RS. At this moment, limited data is available on sarcopenia and its associations with (recovery of) physical activity and -functioning, especially after oncologic surgery. Van Ancum et al. compared muscle mass at hospital admission in 373 older adults (>70 years) to its relation with being at risk of geriatric conditions [41]. Skeletal muscle mass (SMM) and appendicular lean muscle mass (ALM) were assessed using a biodex scan, which measures bioelectrical impedance. In males only, SMM and ALM were significantly associated with an increased risk of functional disability using a risk scoring system for independently functioning in daily activities. They did not find an association in female patients [41]. One cohort study investigates sarcopenia in older patients, including older adults undergoing colorectal or emergency abdominal surgery, and changes in physical performance with follow-up at 13 weeks after admission, but the results have not been published yet [42]. To our knowledge, no other studies have published on sarcopenia and (recovery of) physical activity in surgical and/or oncological patients.

We did not find an association between preoperative RS and postoperative length of hospital stay and complication rates. In contrast, Peng et al. found an association between RS and the risk of major complications and prolonged hospital stays in colorectal liver metastasis surgery [6]. In 423 patients who underwent laparoscopic surgery for colon cancer, an association between RS, complication rates and severe complications was found. Also, Schneider et al. studied 325 patients and found muscle quality on CT-scans, skeletal muscle radiation attenuation (SMRA), as a weak predictor for complications and length of hospital stay after oncologic colon surgery, while muscle mass and quantity were not predictive [43]. In contradiction, another study including 209 patients undergoing laparoscopic colorectal cancer surgery found no association between radiological sarcopenia and overall complication rates [8]. There larger cohorts suggest RS might be a predictor of adverse postoperative outcomes such as complications and length of hospital stay, however, results are contradictive. It should be noted that these studies all use different cut-off values for RS, which makes comparison between the studies more difficult. Also, several options to radiologically assess sarcopenia are used. In recent literature, the TPI has been found predictive when assessing short-term survival prognosis in renal cell carcinoma patients [44]. Radiological-measured psoas muscle mass has also been shown able to predict performance on cardio-pulmonary exercise testing (CPET) in patients undergoing major colorectal surgery [45]. Psoas muscle mass therefore seems a valuable predictor in (surgical) cancer patients. Assessing psoas muscle mass has some disadvantages too, as it is only one muscle, which might be different to assess on CT-scans. Despite, the m. psoas is important in core stability and therefore physical function. In addition, Weerink et al. found a high intra/interobserver correlation in their study assessing psoas muscle mass as predictor of outcomes in surgical solid cancer patients, conform other studies in literature [17,46]. However, there still is no consensus about which measurement is the best for radiological sarcopenia [47]. A validated method of measurement and cut-off value for RS increases comparability between studies and with that, we can better understand the predictive value of sarcopenia for adverse postoperative outcomes in oncologic surgery.

Some strengths and limitations need to be considered when interpreting the findings of this explorative study. A strength of the present study is that the cohort was well defined. Physical activity is objectively measured using accelerometers, whereas most studies use self-reported measurements of physical activity and/or functioning only. Physical activity is measured consecutively for three months, and compared to the patients’ own baseline, therefore objectively providing insight into trends in the recovery of physical activity. A limitation is that the patients in this study might have achieved higher step counts because they were possibly motived by the feedback of their Fitbit devices and were contacted if their step count dropped below 1000 steps/day [10]. In previous non-surgical studies, participants were likely to increase their physical activity levels by more than 25% after they started to wear an accelerometer-based wearable monitor [10,48]. Another strong point is that the presence of RS was assessed using CT imaging, which is considered a golden standard for assessing muscle mass together with MRI [28]. Identifying sarcopenia on a CT scan routinely obtained in the oncological workup does not require additional tests or interventions for the patient [17]. A limitation of RS is that based on functional sarcopenia definitions, such as the European Working Group on Sarcopenia in Older People (EWGSOP) diagnostic algorithm for sarcopenia, stratification for sarcopenia might be different compared to stratification based on RS, and might partially explain why no comparable associations between preoperative sarcopenia and physical activity were found. A final strong point is that this study is first providing insight in preoperative RS and postoperative objectively measured recovery of physical activity. A limitation is the relatively small number of patients included in this explorative design study. Patients participating in this study might have had fewer co-morbidities than patients who refused to participate or for whom surgical treatment did not seem to fit [29]. Also, there might be a selection bias in physical activity as patients with walking aids were excluded from participation, and patients were only analyzed if they were compliant with data synchronization [10]. This selection bias might also explain why the mean age is relatively young compared to other cohorts assessing older adults undergoing cancer surgery [10]. Another limitation in this small cohort is that sarcopenia might manifest differently in sexes. Stratification for sex was not possible in this small cohorts, but different cut-off values per sex were used.

To be able to draw conclusions on the predictive value of (radiological) sarcopenia for physical recovery after oncologic surgery, more data is needed. This exploratory study provides a first insight. The hypothesis that preoperative RS is related to diminished abilities to physically recover after surgery was not confirmed in our data. Future studies should evaluate whether there is an association between functional definitions of sarcopenia and recovery of physical activity. Also, it would be interesting to find out whether preoperative sarcopenia is associated with physical recovery after three months, as our results implicate that it may take a long time after oncological surgery to return to baseline PA. When, in future studies, using steps/day as outcome, what should be noted prior to power analysis, as the spread of steps/day is broad (IQR range 1287−6810 and 3362−5329 for steps/day 3 months postoperatively), large groups might be needed to confirm or discard hypothesis.

## 5 Conclusions

This explorative cohort study in a well-defined cohort of older adults undergoing surgery for solid cancers provides data regarding the association between radiological sarcopenia and recovery of objectively measured physical activity. As no association between radiological sarcopenia and recovery of physical activity was found, radiological measurements of sarcopenia might not be a useful predictor for recovery of physical activity. This underlines the value of assessment of different geriatric domains in older surgical patients. To our knowledge, this is the first explorative study assessing the association between RS and recovery of objectively measured PA. As this is a small cohort, future research in larger cohorts should further investigate this association, and focus on clinical definitions of sarcopenia in oncologic patients and assess whether preoperative (radiological) sarcopenia has a predictive value in postoperative functional outcomes, in addition to clinical outcomes.

## CRediT authorship contribution statement

**Sharon Hendriks:** Conceptualization, Methodology, Validation, Formal analysis, Investigation, Data curation, Writing – original draft, Writing – Review & editing, Visualization. **Monique Huisman:** Methodology, Formal analysis, Writing – review & editing. **Linda Weerink:** Methodology, Resources, Writing – review & editing. **Leonie Jonker:** Conceptualization, Metholodogy, Data curation, Project administration, Writing – review & editing. **Barbara van Munster:** Methodology, Writing – review & editing. **Jacco de Haan:** Methodology, Writing – review & editing. **Geertruida de Bock:** Conceptualization, Methodology, Writing – review & editing. **Barbara van Leeuwen:** Conceptualization, Methodology, Writing – review & editing.

## Conflict of interests

The authors declare that they have no known competing financial interests or personal relationships that could have appeared to influence the work reported in this paper.

## Glossary

Sarcopenia: the loss of muscle mass and strength
Radiological sarcopenia: radiologically diagnosed loss of muscle mass and strength
Moderate to Vigorous Physical Activity (MVPA): higher intensity levels of physical activity defined as the minutes per day spent on activities with an intensity of ≥3 Metabolic Equivalent of Tasks
Metabolic Equivalent of Tasks (MET): Ratio of metabolic rate compared to the resting metabolic rate. The Resting metabolic rate (1 MET) is defined as 1 kcal/kg/hour or the oxygen cost, where the resting metabolic rate is defined as 3.5 ml/kg/min, and is equivalent to the energy cost of sitting quietly
Total Psoas Area (TPA): Calculation of the surface area of both psoas muscles at a certain intersection at imaging (usually at CT-scans or MRI)
Total Psoas Index (TPI): Total Psoas Area adjusted for the patient’s length
Total Psoas Radiological Density (TPRD): A definition of the psoas muscle quality assessed with the radiodensity on CT-scans
Skeletal Muscle Mass (SMM): total mass of all skeletal muscles
Appendicular Lean Muscle Mass (ALM): total mass of skeletal muscles in the upper- and lower extremities
Skeletal muscle radiation attenuation (SMRA): A measurement of muscle density on CT-scans, lower values reflect increased muscle lipid content
