# Supplementary material for: The relation between preoperative radiological sarcopenia and postoperative recovery of physical activity in older surgical cancer patients; an explorative study
Source: J Nutr Health Aging. 2024 Aug 24;28(10):100345. doi: 10.1016/j.jnha.2024.100345 (PMC12877267; doi:10.1016/j.jnha.2024.100345)
Supplement: Supplementary file 1 [file mmc1.docx]

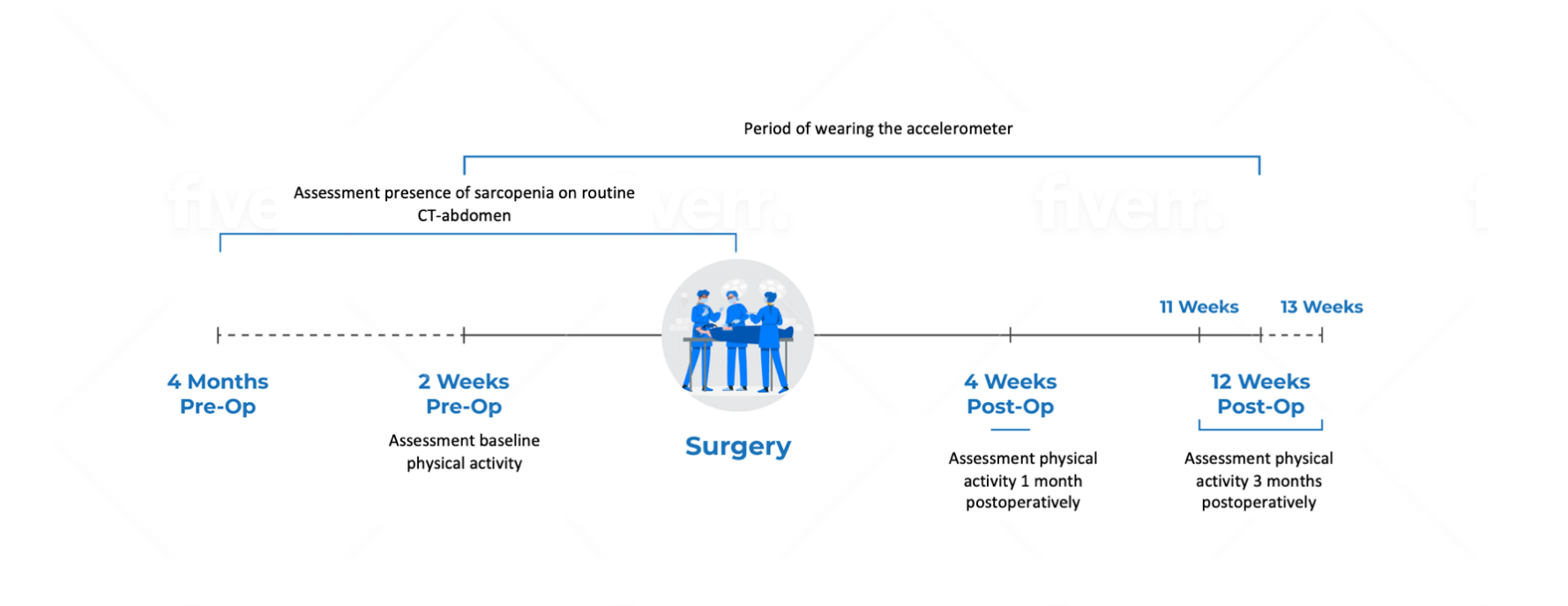
**Supplementary figure 1**: Schematic timeline of measurements in this study.


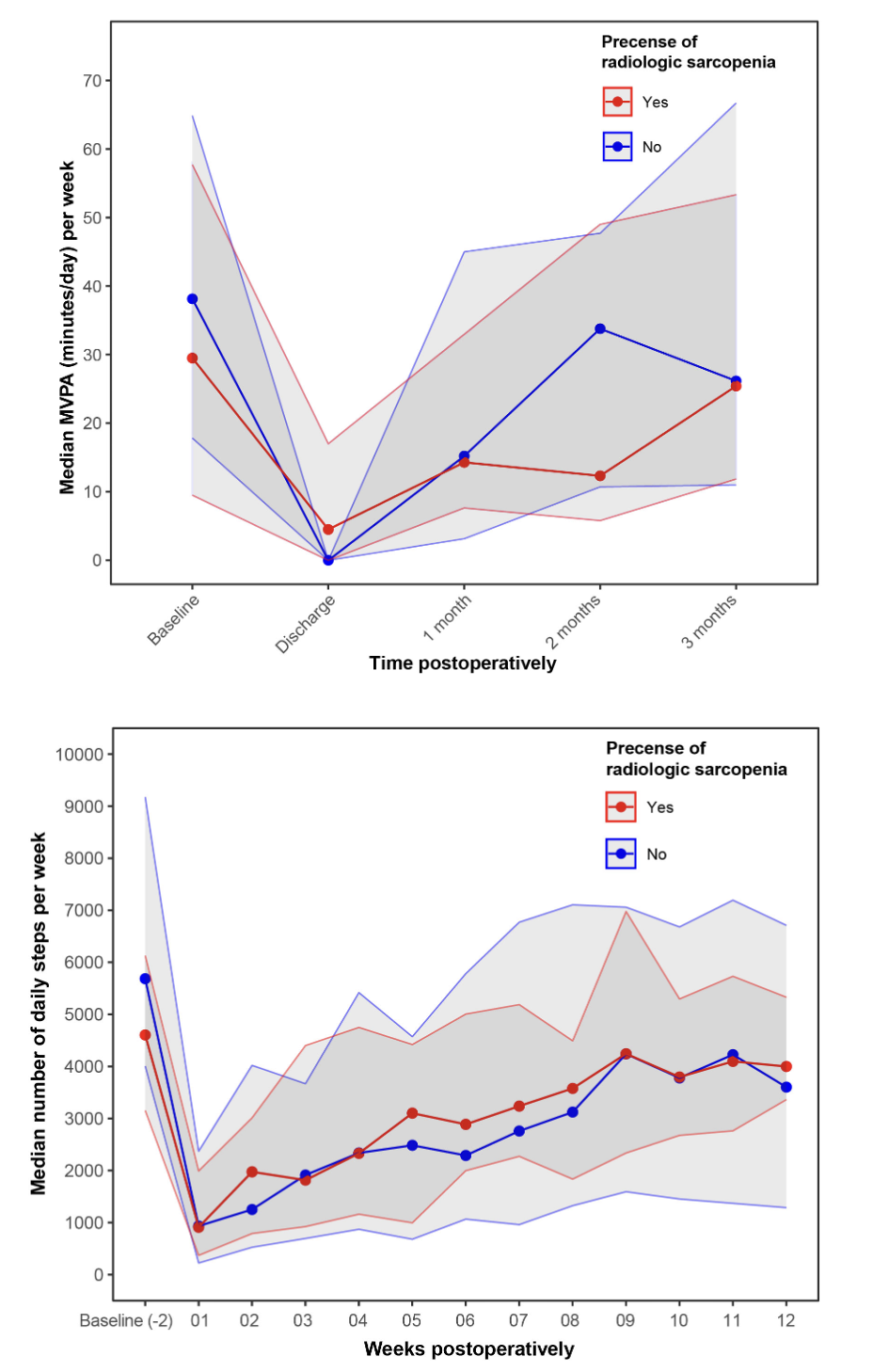


**Supplementary figure 2**: Median steps/day per week for patients with- and without radiologic sarcopenias. Lines without dots represent the 95% confidence interval.

| **Supplementary Table 1 Physical activity pre- and postoperatively stratified by the absence or presence of radiological sarcopenia in intra-cavitary surgical patients only (n=39)** | | | |
| --- | --- | --- | --- |
| **Steps per day:** median [IQR] | | | |
|  | **Non-radiologic**  **sarcopenic** (n=23) | **Radiologic Sarcopenic** (n=16) | **p-value**^+^ |
| **Baseline** | 5403 [4469-7433] | 4736 [2953-6613] | 0.53 |
| **At 1 month* postoperatively** | 1967 [840-4942] | 2723 [1116-5162] | 0.77 |
| **At 3 months*** | 3395 [1287-6108] | 4587 [3362-7478] | 0.39 |
| **% of baseline at 3 months*** | 79.9 [50.4-100.9] | 88.0 [65.6-134.2] | 0.07 |
| **Recovered**^#^ **at 3 months**   - Yes - No | 8 (38.1%)  13 (61.9%) | 7 (46.7%)  8 (53.3%) | 0.45 |
| **MPVA in minutes/day**, median [IQR]) | | | |
| **baseline** | 37 [17-60] | 30 [10-58] | 0.68 |
| **MVPA 1 month*** | 15 [3-35] | 14 [8-33] | 0.70 |
| **MVPA 3 months*** | 26 [11-55] | 25 [12-53] | 0.89 |
| **% of baseline at 3 months*** | 69.8 [44.6-126.3] | 88.1 [51.6-147.1] | 0.43 |
| **Recovered**^#^ **at 3 months**   - Yes - No | 6 (30.0%)  14 (70.0%) | 5 (45.5%)  6 (54.5%) | 0.74 |

Legend table 2: *IQR* Interquartile range; *MVPA* Moderate – Vigorous Physical Activity.

*postoperatively, ^#^recovered = >90% of baseline value.

^+^Values for patients with- and without radiologic sarcopenia were testes using Mann Whitney U Tests and Chi Square where appropriate.
